# Balancing Radiation Dose and Image Quality: Protocol Optimization for Mobile Head CT in Neurointensive Care Unit Patients

**DOI:** 10.3390/diagnostics16020256

**Published:** 2026-01-13

**Authors:** Damian Mialkowskyj, Robert Stahl, Suzette Heck, Konstantinos Dimitriadis, Thomas David Fischer, Thomas Liebig, Christoph G. Trumm, Tim Wesemann, Robert Forbrig

**Affiliations:** 1 Institute for Diagnostic and Interventional Neuroradiology, University Hospital, LMU Munich, Marchioninistr. 15, 81377 Munich, Germany; damian.mialkowskyj@med.uni-muenchen.de (D.M.); robert.stahl@med.uni-muenchen.de (R.S.); thomas.fischer@med.uni-muenchen.de (T.D.F.); thomas.liebig@med.uni-muenchen.de (T.L.);; 2 Department of Neurology, University Hospital, LMU Munich, Marchioninistr. 15, 81377 Munich, Germany; suzette.heck@med.uni-muenchen.de (S.H.); konstantin.dimitriadis@med.uni-muenchen.de (K.D.); 3 Radiologie Augsburg Friedberg ÜBAG, Hermanstraße 15, 86150 Augsburg, Germany

**Keywords:** brain, image quality, mobile computed tomography, neurocritical care, radiation exposure

## Abstract

**Objective:** Mobile head CT enables bedside neuroimaging in critically ill patients, reducing risks associated with intrahospital transport. Despite increasing clinical use, evidence on dose optimization for mobile CT systems remains limited. This study evaluated whether an optimized CT protocol can reduce radiation exposure without compromising diagnostic image quality in neurointensive care unit patients. **Methods:** In this retrospective single-center study, twenty-two non-contrast head CT examinations were acquired with a second-generation mobile CT scanner between March and May 2023. Patients underwent either a default (group A, *n* = 14; volumetric computed tomography dose index (CTDI_vol_) 44.1 mGy) or low-dose CT protocol (group B, *n* = 8; CTDI_vol_ 32.1 mGy). Regarding dosimetry analysis, we recorded dose length product (DLP) and effective dose (ED). Quantitative image quality was assessed by manually placing ROIs at the basal ganglia and cerebellar levels to determine signal, noise, signal-to-noise ratio, and contrast-to-noise ratio. Two neuroradiologists independently rated qualitative image quality using a four-point Likert scale. Statistical comparisons were performed using a significance threshold of 0.05. **Results:** Median DLP and ED were significantly lower for group B (592 mGy·cm, 1.12 mSv) than for group A (826 mGy·cm, 1.57 mSv; each *p* < 0.0001). Quantitative image quality parameters did not differ significantly between groups (*p* > 0.05). Qualitative image quality was rated excellent (median score 4). **Conclusions:** The optimized mobile head CT protocol achieved a 28.7% reduction in radiation exposure while maintaining high diagnostic image quality. These findings support the adoption of low-dose strategies in mobile CT imaging in line with established radiation protection standards.

## 1. Introduction

Computed tomography (CT) is essential for the diagnosis and monitoring of neurointensive care unit (neuro-ICU) patients suffering from critical conditions such as traumatic brain injury, ischemic stroke, spontaneous intracerebral hemorrhage, or aneurysmal subarachnoid hemorrhage [1,2,3]. These patients frequently require repeated imaging to assess disease progression, detect complications such as hydrocephalus resulting in elevated intracranial pressure, and guide clinical management. Bedside imaging using mobile CT systems offers a substantial clinical advantage by eliminating the risks associated with intrahospital transport—including hemodynamic instability and elevations in intracranial pressure—and by reducing procedural delays and logistical complexity [4,5,6,7].

The feasibility and diagnostic performance of earlier generations of mobile CT systems have been demonstrated in several clinical studies [6,7]. More recently, second-generation mobile CT systems have introduced advanced detector technology [8] and modern iterative reconstruction (IR) algorithms [9], resulting in improved spatial resolution and image quality that approach those of stationary systems [10,11]. These technological advancements enable reliable bedside imaging even in patients with limited physiological stability, allowing repeated examinations without the need for patient transfer [6,7,10,11].

Despite the clear logistical and patient safety advantages of mobile CT, systematic investigations focusing on radiation dose optimization in this setting remain limited. Most recent studies have primarily addressed clinical feasibility and workflow efficiency rather than dose reduction [10,11]. Standard head CT protocols for second-generation mobile CT systems typically employ a volumetric CT dose index (CTDI_vol_) between 40 and 50 mGy to ensure sufficient signal-to-noise ratio (SNR) [12] and contrast-to-noise ratio (CNR) [10,11], whereas modern stationary CT systems, such as third-generation dual-source CT, can achieve high image quality at CTDI_vol_ values of approximately 35 mGy [13].

Given that second-generation mobile CT systems are now also equipped with improved detectors and IR techniques, further reduction in tube output without compromising image quality appears feasible even in the bedside setting [8,9,14,15,16,17,18]. For stationary CT imaging, Christe et al. demonstrated that the use of IR and advanced detector technology allows dose reductions of up to 70% compared with filtered back projection while preserving diagnostic image quality [8]. Similarly, Guziński et al. showed that low-dose head CT protocols using IR can improve image quality in the posterior fossa [14], and Chen et al. achieved a 50% dose reduction in head and neck CT angiography using low tube voltage and IR while maintaining diagnostic quality [15].

These technological developments are consistent with the ALARA (as low as reasonably achievable) principle and align with current guidelines of the German Federal Office for Radiation Protection (BfS), which recently updated the diagnostic reference level (DRL) for head CT to a CTDI_vol_ of 55 mGy [19]. Nevertheless, evidence-based dose optimization strategies for mobile CT systems remain scarce.

Therefore, the present monocentric study investigated an unselected cohort of neuro-ICU patients examined with a second-generation mobile head CT scanner, either before or after protocol adjustment, to evaluate the impact of dose reduction in the bedside setting. In addition to dosimetric analysis, we performed a comprehensive comparison of quantitative and qualitative image quality metrics to determine whether dose optimization can be achieved without compromising diagnostic performance.

## 2. Materials and Methods

### 2.1. Study Design and Ethical Approval

This retrospective, single-center study was conducted at the Institute of Neuroradiology, LMU Klinikum München, Campus Großhadern. The study was performed in accordance with the principles of the Declaration of Helsinki and approved by the Ethics Committee of Ludwig-Maximilians-University (Project No. 25-1058-KB; date of approval: 4 December 2025). All data were anonymized before analysis. The requirement for individual informed consent was waived because all examinations were clinically indicated.

### 2.2. Patient Population

The second-generation mobile CT scanner (SOMATOM On.site, Siemens Healthineers, Forchheim, Germany; Software Version VB60A) [10,11] was provided by the manufacturer and used at the neuro-ICU between March and May 2023. Initially, the manufacturer’s default head CT protocol was applied (group A, March–April 2023; CTDI_vol_ = 44.1 mGy, *n* = 14), after which the protocol was switched to a low-dose setting (group B, May 2023; CTDI_vol_ = 32.1 mGy, *n* = 8).

Inclusion criteria: Adult patients (≥18 years) admitted to the neuro-ICU with an indication for cranial CT imaging, including acute ischemic stroke, intracerebral hemorrhage, subarachnoid hemorrhage, or postoperative monitoring after neurosurgical procedures.

Exclusion criteria: Incomplete dose documentation, major postoperative skull defects, metallic implants causing non-correctable streak artifacts, or severe motion artifacts that prevented image analysis.

### 2.3. CT Acquisition Protocol

All brain imaging was performed using the SOMATOM On.site mobile CT scanner. The system integrates a self-shielded gantry and a Stellar detector design with integrated analog-to-digital conversion at the detector level, reducing electronic noise and improving image quality at lower exposure levels [8]. Details of the CT acquisition parameters for both groups are shown in Table 1.

No intravenous contrast medium was administered. Scans were performed with patients in the supine position, with the head immobilized using the integrated cradle support. All images were reconstructed in axial orientation and archived in the institutional picture archiving and communication system (PACS).

### 2.4. Radiation Dose Assessment

Radiation dose parameters were extracted from the scanner-generated DICOM dose reports and included CTDI_vol_ as well as dose-length product (DLP). The effective dose (ED) was calculated according to the method described by Deak et al. [20], using the region-specific conversion factor for adult head CT:

ED=DLP×0.0019

This conversion factor (k = 0.0019 mSv·mGy^−1^·cm^−1^) corresponds to the recommendations of the International Commission on Radiological Protection ICRP Publication 103 [21] and is widely used in contemporary CT dosimetry. The local DRL (CTDI_vol_) was defined as the 75% percentile of the dose distribution [22].

### 2.5. Objective Image Quality Analysis

Quantitative image quality was assessed using region-of-interest (ROI)-based measurements, following the methodology of Christe et al. [8] and Guziński et al. [14]. In addition, the ROI placement strategy and quantitative evaluation approach were aligned with the framework described by Forbrig and colleagues [23], who demonstrated the reproducibility and diagnostic relevance of ROI-based SNR and noise measurements in head CT.

In detail, circular ROIs with a size of 20–30 mm^2^ were manually placed bilaterally within pre-defined, normal-appearing anatomical regions of both the telencephalon and cerebellum. In these regions, gray matter (GM) and white matter (WM) were differentiated using separate ROIs, allowing for a consistent assessment of image noise and contrast. The specific ROI locations were defined as follows:

- GM telencephalon: head of the caudate nucleus, lentiform nucleus and/or thalamus;
- WM telencephalon: internal capsule;
- GM cerebellum: cortex of the cerebellar hemisphere;
- WM cerebellum: level of the middle cerebellar peduncle.

Mean signal intensity (Hounsfield units (HU); i.e., signal) and standard deviation (SD; i.e., noise) were recorded for each ROI using a soft-tissue window (width 80 HU, level 40 HU). SNR and CNR were calculated as:

$$SNR=\frac{MeanHU}{SD}$$

$$CNR=\frac{\left|{MeanHU}_{GM}-{MeanHU}_{WM}\right|}{{SD}_{WM}}$$

All measurements were performed by a neuroradiologist (R.F.) with more than 13 years of experience in diagnostic neuroradiology using the institutional PACS measurement tools. Values were averaged for both hemispheres to minimize variability.

### 2.6. Subjective Image Quality Analysis

Subjective image quality assessment was independently performed in random order by two board-certified neuroradiologists. The readers were blinded to the two groups, and window settings were freely adjustable. Image quality was rated using a four-point Likert scale (Table 2), adapted from Guziński et al. [14] and Chen et al. [15]:

The following features in normal-appearing regions were evaluated: gray–white matter differentiation, visibility of basal ganglia, ventricular and posterior fossa structures, subjective image noise, and the presence of streak or beam-hardening artifacts caused by bone structures.

### 2.7. Statistics

The distribution of age, radiation dose metrics, and quantitative image quality values in both groups was initially assessed for normality using visual inspection of histograms and the Shapiro–Wilk test. Since not all data were normally distributed, values are reported as median [25th, 75th percentiles]. Between-group differences were evaluated using the Mann–Whitney U test. Analysis was performed using R (R Core Team (2023). R: A Language and Environment for Statistical Computing. R Foundation for Statistical Computing, Vienna, Austria). A two-sided alpha level of 0.05 was used to define statistical significance throughout the study.

## 3. Results

A total of 22 bedside head CT examinations were included, comprising 14 scans acquired with the standard protocol (63.6%, group A) and 8 scans with the optimized low-dose protocol (36.4%, group B). Of the 22 neuro-ICU patients, 10/22 (45.5%) presented with ischemic stroke, 5/22 (22.7%) with aneurysmal subarachnoid hemorrhage treated by endovascular aneurysm occlusion in 4 cases and by aneurysm clipping in 1 case, 3/22 (13.6%) with spontaneous intracerebral hemorrhage, 3/22 (13.6%) with traumatic brain injury, and 1/22 (4.5%) with neurosarcoidosis. 14/22 patients (63.6%) were male and 8/22 (36.4%) female; the median age was 53 years (range, 28–89 years).

### 3.1. Radiation Dose

**Figure 1:**
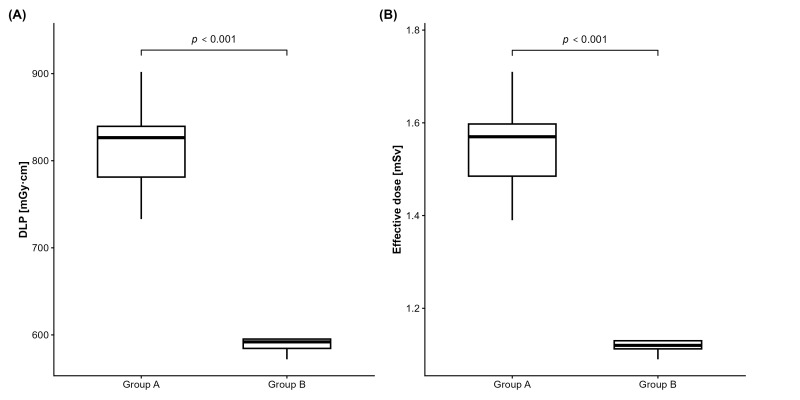
Radiation dose metrics before (group A) and after dose optimization (group B). Both DLP (**A**) and effective dose (**B**) were significantly lower in group B after reduction in CTDI_vol_ from 44.1 mGy to 32.1 mGy. CTDI_vol_, volumetric computed tomography dose index; DLP, dose length product.

Radiation dose metrics demonstrated the expected reduction in exposure with the optimized protocol (group A CTDI_vol_ 44.1 mGy, group B CTDI_vol_ 32.1 mGy). In detail, median values of DLP and ED were 826.5 [781.3, 839.5] mGy·cm and 1.57 [1.48, 1.60] mSv for group A, and 592 [584.5, 595.5] mGy·cm and 1.12 [1.11, 1.13] mSv for group B (*p* < 0.0001, each; Figure 1), yielding a relative dose reduction of 28.7% in the latter group. Scan length was statistically similar for both groups (median 162 versus 159 mm, *p* = 1.000).

### 3.2. Objective Image Quality

Results of the quantitative image quality assessment for the telencephalon (basal ganglia level) and cerebellum are summarized in Table 3. Across all measured locations, group-wise distributions were closely comparable, and none of the metrics showed a statistically significant difference between the standard (group A) and dose-reduced CT protocol (group B) (*p* > 0.05, each).

**Figure 2:**
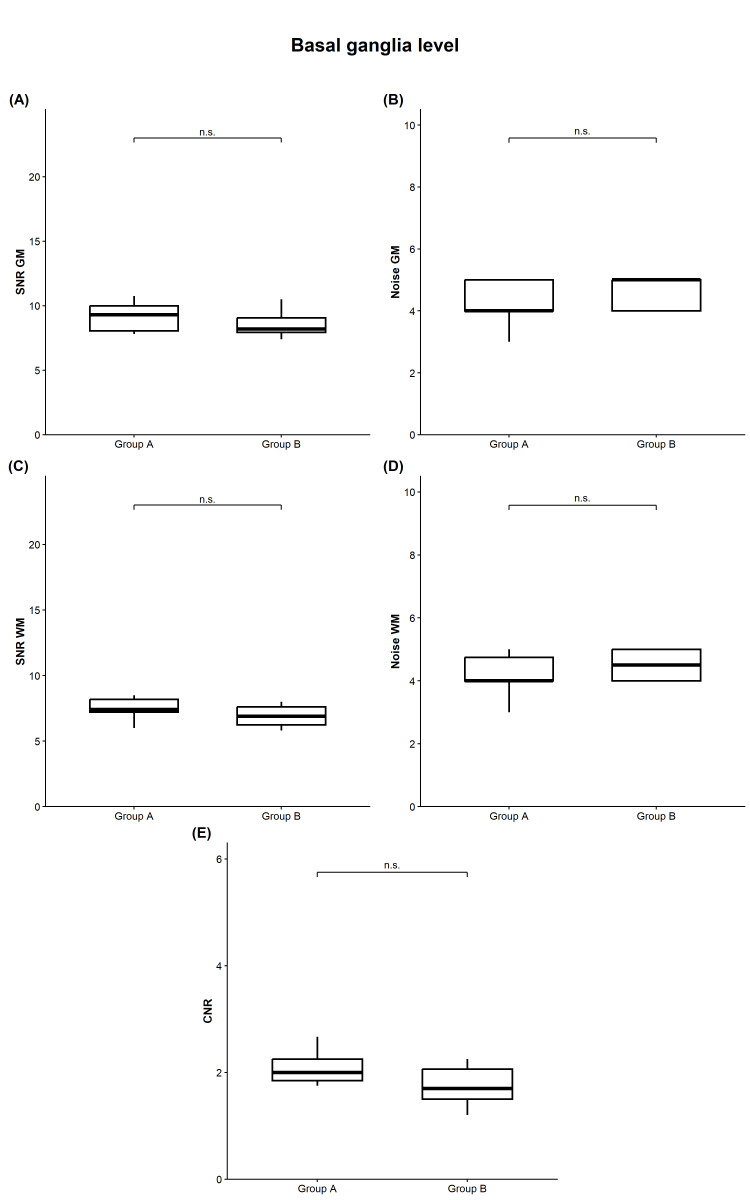
Quantitative image quality at the basal ganglia level. Comparison of group A (standard dose protocol) and group B (optimized dose protocol) neither showed significant differences in SNR and image noise within gray matter (**A**,**B**) nor white matter (**C**,**D**) (*p* > 0.05, each). Also, CNR values were not significantly different despite minor decrease in group B ((**E**), *p* > 0.05). CNR, contrast-to-noise ratio; GM, gray matter; n.s., not significant; SNR, signal-to-noise ratio; WM, white matter.

At the level of the basal ganglia (Figure 2), the median GM signal was identical between both protocols (40 HU, each; *p* = 0.833). Median GM noise was slightly lower for group A compared to group B (4.00 versus 5.00, *p* = 0.354). Consequently, we documented a minor decrease in median GM SNR in group B without reaching statistical significance (9.30 versus 8.20, *p* = 0.257).

WM signal at the basal ganglia level was likewise comparable, with medians of 31.50 HU in group A and 31.00 HU in group B (*p* = 0.755). Median WM noise was 4.00 for group A versus 4.50 for group B (*p* = 0.286), yielding slightly higher SNR in group A (median 7.40 versus 6.90, *p* = 0.245).

Median CNR at this level was 2.00 for group A and 1.70 for group B (*p* = 0.148), again indicating no statistically significant reduction in tissue contrast when using the low-dose CT protocol.

**Figure 3:**
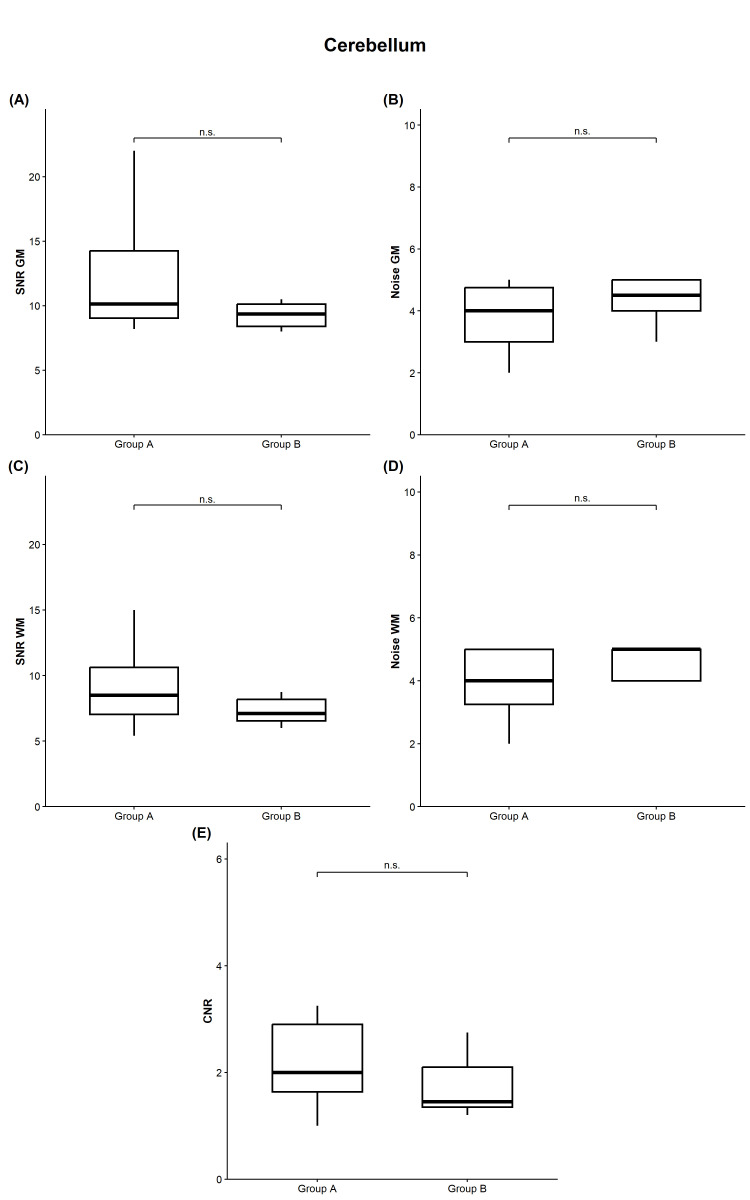
Quantitative image quality in the cerebellum. Comparison of group A (standard dose protocol) and group B (optimized dose protocol) revealed no significant differences in SNR and image noise within gray matter (**A**,**B**) and white matter (**C**,**D**), respectively (*p* > 0.05, each). CNR was slightly lower in group B without reaching statistical significance ((**E**), *p* > 0.05). CNR, contrast-to-noise ratio; GM, gray matter; n.s., not significant; SNR, signal-to-noise ratio; WM, white matter.

In the cerebellum (Figure 3), the median GM signal was 41.50 HU for both groups (*p* = 0.678). Median GM noise values were 4.00 for group A and 4.50 for group B (*p* = 0.208). Consequently, we recorded a median GM SNR of 10.12 in group A and 9.35 in group B (*p* = 0.161).

Cerebellar WM signal showed medians of each 34.00 HU (*p* = 0.863), while median WM noise was 4.00 in group A and 5.00 in group B (*p* = 0.131). Corresponding median WM SNR was 8.50 (group A) and 7.10 (group B) (*p* = 0.218), respectively.

Regarding cerebellar CNR, we calculated a median of 2.00 for group A compared with 1.45 for group B (*p* = 0.217), with overlapping interquartile ranges and no statistically significant difference between protocols.

### 3.3. Subjective Image Quality

**Figure 4:**
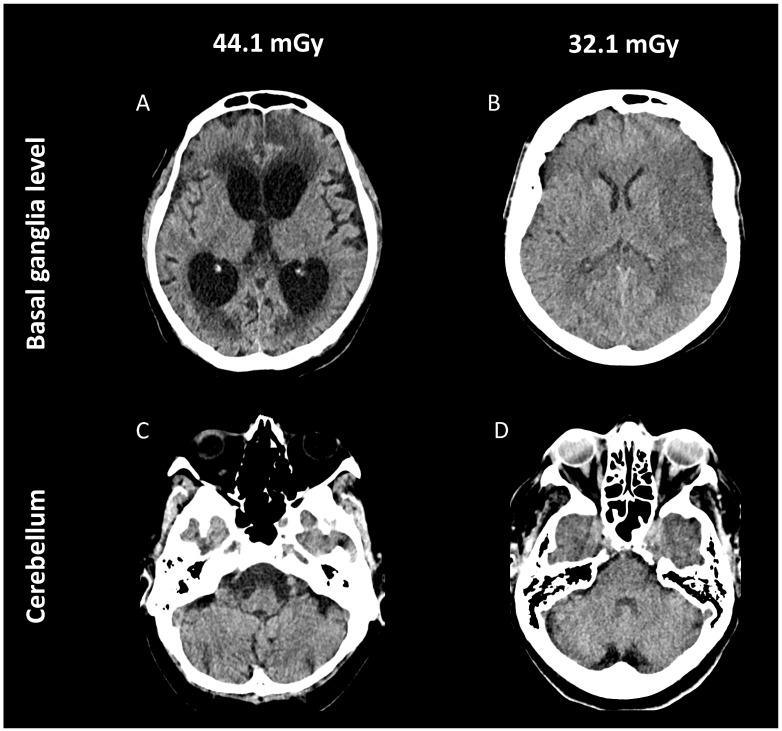
Illustrative cases of mobile head CT before (**A**,**C**) and after dose optimization (**B**,**D**). (**A**,**C**) Example of a 54-year-old male patient admitted to the neuro-ICU due to aneurysmatic subarachnoid hemorrhage ten days ago. Images show communicating post-hemorrhagic hydrocephalus with periventricular CSF diapedesis and hyperdense blood residuals at the anterior cingulate gyrus region on both sides. Visibility of gray-white differentiation, basal ganglia, and infratentorial structures is very good, while only minimal image noise and streak-/beam-hardening artifacts are noted (rating 4, excellent). (**B**,**D**) Example of a 61-year-old female patient with history of left M1-MCA occlusion on the day before. The consecutive acute infarction with territorial loss of gray-white differentiation at the left fronto-insular and temporal region is clearly visible, while the medial basal ganglia are not involved. CSF compartments and infratentorial structures are precisely depicted; image noise and streak-/beam-hardening artifacts are negligible (rating 4, excellent). CSF, cerebrospinal fluid; CT, computed tomography; neuro-ICU, neurointensive care unit; M1-MCA, M1 segment of the middle cerebral artery.

Examples of subjective image quality evaluation are shown in Figure 4. Regarding blinded assessment of images, the median rating of two readers was 4 (excellent) for both groups, thus statistically equal (*p* > 0.05, each).

## 4. Discussion

In this retrospective single-center study, we show that radiation exposure in bedside mobile head CT examinations of neuro-ICU patients can be reduced by almost one third without a measurable loss of image quality. The optimized low-dose protocol with a CTDI_vol_ of 32.1 mGy (standard protocol: 44.1 mGy) resulted in significantly lower median DLP and ED values (592 vs. 826 mGy·cm; 1.12 vs. 1.57 mSv), while quantitative and qualitative image quality metrics remained statistically stable in both supratentorial and infratentorial regions.

With regard to the literature, dosimetry values obtained before dose reduction in this mobile head CT study were comparable to those reported by Goertz and colleagues, who applied the same second-generation mobile CT scanner with identical default protocol settings (mean DLP 939 ± 132 mGy·cm) [10]. After dose optimization, our values were similar to recently published data by Forbrig and colleagues, who analyzed dosimetrics of an extended stroke protocol using stationary single- and dual-source CT systems, reporting median DLP values of 517–683 mGy·cm and median ED values of 0.78–1.09 mSv for unenhanced head CT [13].

Furthermore, dosimetry values of the present study were clearly lower (both before and after protocol adjustment) than those reported for stationary head CT by Menon and colleagues in 2015 (2.0 mSv) [24] and Wu and colleagues in 2020 (1.83 mSv) [25]. From a radiation protection perspective, both head CT protocols used in this study complied with national DRLs in Germany (55 mGy) [19] as well as those reported for several Commonwealth countries and the USA (47–82 mGy) [26].

Quantitative image assessment revealed no substantial differences between the applied protocols. At the basal ganglia level, gray matter (GM) and white matter (WM) signal, noise levels, SNR, and CNR were highly comparable, with overlapping interquartile ranges and *p*-values between 0.148 and 0.833. These findings indicate that lowering CTDI_vol_ from 44.1 to 32.1 mGy did not result in statistically significant degradation of GM/WM contrast or noise characteristics in supratentorial structures. Median SNRs and CNRs before dose reduction were comparable to those reported by Goertz et al. [10] (our study: SNR GM/WM 9.3/7.4, CNR 2.0; their study: SNR GM/WM 7.8/7.1, CNR 2.5). Median image noise at this level was also comparable (our study: 4.0/4.0; their study: 5.2/4.7), while the noise slightly increased after dose reduction (GM/WM: 5.0/4.5) without reaching statistical significance.

At the cerebellar level, no significant differences were observed between groups despite a minor increase in median image noise (GM/WM 4.0/4.0 vs. 4.5/5.0) and a slight decrease in median SNR (GM/WM 10.1/8.5 vs. 9.4/7.1) and CNR (2.0 vs. 1.5) after dose reduction. Notably, independent of the applied protocol, cerebellar noise levels (median 4.0–5.0) were comparable to those reported by Andersson et al., who used the same mobile CT scanner and default settings [11]. Contemporary stationary CT systems (e.g., dual-source, spectral, or photon-counting CT) may achieve even higher SNRs (up to 20) and CNRs (up to 4) [10,11,13,27,28,29,30], owing to further reductions in image noise to values of 2–3.

Importantly, qualitative image assessment demonstrated that the modest numerical increase in noise levels as well as reductions in SNR and CNR observed with the low-dose protocol did not translate into a subjective loss of diagnostic confidence. Both blinded neuroradiologists rated overall image quality as excellent (median score 4), irrespective of the applied protocol. In contrast to previous reports [10,11], we did not observe a substantial deterioration of image quality in the posterior fossa, either quantitatively or qualitatively. Notably, no relevant beam-hardening or streak artifacts caused by the skull were observed, and image noise was rated equally low for either protocol.

These findings highlight the substantial technological advances in CT scanner development over the past decades. Although the concept of mobile head CT is not new, early systems failed to achieve widespread clinical adoption due to high acquisition costs and limited image quality [31,32]. For instance, the Philips Tomoscan M, one of the first portable CT scanners, generated images that were inferior to those of contemporaneous stationary systems, mainly because of increased image noise, a bulky design, and limited technical reliability in the ICU setting [31]. Likewise, Abdullah et al. demonstrated that the CereTom CT scanner introduced in 2004 provided significantly lower image quality than stationary CT systems, particularly with respect to radiation artifacts, GM/WM differentiation, and intracranial lesion delineation [32].

In contrast, second-generation mobile CT systems equipped with advanced detector designs and iterative reconstruction algorithms [8,9,10,11] offer a relevant safety margin in conventional dose settings that allows for dose reduction without meaningful compromise of diagnostic performance, despite previous reports of slightly inferior overall image quality and increased noise levels compared with stationary CT systems [10,11]. However, both cited studies attempted to match the CTDI_vol_ for mobile and stationary scanners without harmonizing important protocol parameters (e.g., pitch, total collimation, voxel size, or reconstruction kernel), which each directly impact the noise level and therefore image quality. A recent phantom study highlighted this issue, showing that the newest version of the mobile CT system applied in this study performed similarly (or sometimes even better) compared to stationary models in low-contrast detectability imaging tasks [12]. This dosimetric aspect is especially important for neuro-ICU patients with severe brain injury, who frequently require repeated CT examinations within short time intervals [1,2,3,4,5,6,7,10,11]. Consequently, reducing the radiation dose per examination directly lowers cumulative exposure and supports adherence to the ALARA principle in this high-risk population.

Several limitations should be acknowledged. First, the sample size was modest (22 patients), which may have limited statistical power and reduced sensitivity for detecting small dose-related changes. For example, aspects such as variability between patients in the two groups may explain the missing statistical significance of noise increase after dose reduction. Second, the study was conducted at a single institution using one specific mobile CT system, limiting generalizability to other manufacturers, hardware, and protocol settings. Third, image quality relied on quantitative metrics and subjective ratings based primarily on normal anatomy, as the patient population was small and heterogeneous; future, larger studies should include diagnostic accuracy for specific pathologies such as early ischemic changes or focal subarachnoid hemorrhage. Fourth, image quality in the presence of metallic implants was not evaluated, as dedicated metal artifact reduction algorithms were not applied. Fifth, we did not perform phantom measurements prior to dose reduction, which precluded a systematic, physics-based optimization of acquisition parameters. As a result, the choice of a reduced CTDI_vol_ of 32.1 mGy was based on a pragmatic and deliberately conservative approach to minimize the risk of impaired image quality. This decision was influenced by a previous report by Andersson et al. [11], in which a substantially lower CTDI_vol_ (24.1 mGy) was applied in a single examination without accompanying image quality assessment. Importantly, image quality at a CTDI_vol_ of 32.1 mGy remained excellent, suggesting that further dose reduction may be feasible and should be explored in future studies using stepwise dose optimization. Finally, workflow-related aspects relevant to point-of-care imaging were not assessed, as the focus of this study was on dosimetry and image quality.

## 5. Conclusions

This study demonstrates that an approximately 29% reduction in radiation dose for mobile head CT can be achieved without measurable loss of image quality. Median DLP and effective dose decreased from 826 mGy·cm and 1.57 mSv (CTDI_vol_ 44.1 mGy) to 592 mGy·cm and 1.12 mSv (CTDI_vol_ 32.1 mGy), while both quantitative and subjective image quality remained high. These results support the systematic implementation of optimized low-dose mobile head CT protocols and underscore the importance of continuous protocol evaluation in light of ongoing technical advances in detector design and iterative reconstruction algorithms, considering mobile CT systems of various manufacturers to enhance generalizability.

## Figures and Tables

**Table 1:** CT acquisition protocol.

Parameter	Default Protocol (Group A)	Optimized Protocol (Group B)
Tube voltage	120 kVp	120 kVp
Tube current time product (eff.)	40 mAs	29 mAs
CTDI_vol_	44.1 mGy	32.1 mGy
Rotation time	1.0 s	1.0 s
Collimation	32 × 0.6 mm	32 × 0.6 mm
Pitch	0.55	0.55
Slice thickness	0.8 mm	0.8 mm
Reconstruction thickness	2 mm/5 mm	2 mm/5 mm
Reconstruction kernel	H31s (soft tissue)	H31s (soft tissue)
IR algorithm	SAFIRE (level 2)	SAFIRE (level 2)
Field of view	260 mm	260 mm
Scan range	Skull base to vertex	Skull base to vertex

CTDI_vol_, volumetric computed tomography dose index; eff., effective; IR, iterative reconstruction; SAFIRE, Sinogram Affirmed Iterative Reconstruction.

**Table 2:** Qualitative image quality rating (4-point Likert scale).

Score	Description
1	Poor (non-diagnostic due to severe noise or artifacts)
2	Moderate (reduced diagnostic confidence)
3	Good (adequate for diagnosis)
4	Excellent (high diagnostic confidence, minimal artifacts)

**Table 3:** Quantitative image quality analysis.

ROI	Group A (*n* = 14)	Group B (*n* = 8)	*p*-Value
Basal ganglia level			
Signal GM	40.00 [39.25, 40.75]	40.00 [38.50, 41.00]	0.833
Noise GM	4.00 [4.00, 5.00]	5.00 [4.00, 5.00]	0.354
SNR GM	9.30 [8.05, 10.00]	8.20 [7.95, 9.06]	0.257
Signal WM	31.50 [30.00, 32.75]	31.00 [29.00, 32.50]	0.755
Noise WM	4.00 [4.00, 4.75]	4.50 [4.00, 5.00]	0.286
SNR WM	7.40 [7.21, 8.19]	6.90 [6.25, 7.62]	0.245
CNR	2.00 [1.85, 2.25]	1.70 [1.50, 2.06]	0.148
Cerebellum			
Signal GM	41.50 [40.25, 43.75]	41.50 [40.00, 42.00]	0.678
Noise GM	4.00 [3.00, 4.75]	4.50 [4.00, 5.00]	0.208
SNR GM	10.12 [9.04, 14.25]	9.35 [8.40, 10.12]	0.161
Signal WM	34.00 [29.25, 35.75]	34.00 [32.00, 35.00]	0.863
Noise WM	4.00 [3.25, 5.00]	5.00 [4.00, 5.00]	0.131
SNR WM	8.50 [7.05, 10.62]	7.10 [6.55, 8.19]	0.218
CNR	2.00 [1.64, 2.90]	1.45 [1.35, 2.10]	0.217

Median values with 25th and 75th percentiles as well as *p*-values are reported for all parameters. CNR, contrast-to-noise ratio; GM, gray matter; *n*, number; ROI, region of interest; SNR, signal-to-noise ratio; WM, white matter.

## Data Availability

The original contributions presented in this study are included in the article. Further inquiries can be directed to the corresponding author.
